# Editorial: Autism: Innovations and Future Directions in Psychological Research

**DOI:** 10.3389/fpsyg.2021.832008

**Published:** 2022-01-17

**Authors:** Emma Gowen, Laura Crane, Christine M. Falter-Wagner

**Affiliations:** ^1^Body, Eye and Movement Lab, Division of Neuroscience and Experimental Psychology, Faculty of Biology, Medicine and Health, Manchester Academic Health Science Centre, University of Manchester, Manchester, United Kingdom; ^2^Centre for Research in Autism and Education (CRAE), UCL Institute of Education, London, United Kingdom; ^3^Department of Psychiatry and Psychotherapy, Medical Faculty, LMU Munich, Munich, Germany

**Keywords:** autism, psychological research, cognition, perception, neuroscience, participatory research

Psychological research on autism has a long tradition, covering multiple fields including cognition, perception, clinical research, neuroscience, and social psychology. This Research Topic brings together the latest research in this area, mapping key developments, innovations, and future directions. In this editorial, we will discuss six themes that we have identified across the 22 contributions to this Research Topic: (1) Theories and mechanisms; (2) Characterization of autism; (3) Sensory experiences, perception and movement; (4) Language; (5) Support and interventions; and (6) Methods and technologies. We also provide thoughts on future directions in the field.

## Theories and Mechanisms

Recent discussions have focused on the double-empathy theory (e.g., Milton, 2012; Bolis et al., 2017; but see Georgescu et al., 2020), which interprets communication “difficulties” associated with autism as a bidirectional breakdown between two interaction partners. Building on this theory, Crompton et al. conducted an innovative empirical study examining interpersonal rapport as a function of the neurology of interaction partners, and the person rating levels of rapport. When rating rapport after semi-structured conversations, homogeneous dyads of non-autistic people reported highest levels of rapport, followed by homogeneous dyads of autistic people and lastly mixed (autistic/non-autistic) dyads. Interestingly, taking an outside perspective, when rating observed rapport between interaction partners, homogeneous dyads of autistic individuals were rated highest concerning observed rapport, followed by homogeneous dyads of non-autistic individuals and lastly, again, mixed (autistic/non-autistic) dyads, supporting the double empathy theory.

Beyond specific aspects of functioning, Gernert et al. suggest that empirical and theoretical considerations should move toward a more comprehensive outlook on autism. The authors' Generalized Adaptation Account suggests potential connections between findings from genetics, neurobiology, endocrinology, cellular and neuronal connectivity levels. In this framework, aberrations of neurodevelopmental signaling pathways link up to alterations of neuronal connectivity with cascading effects on neuroendocrine dysregulations and impact on circadian functioning. Consequently, chronic distress and hyperactivation of the hypothalamus-pituitary-adrenal (HPA)-axis result in oxytocinergic downregulation linked to social functioning. This unifying account tries to capture both the complexity of presentation of autism and, in particular, its heterogeneity.

## Characterization of Autism

Two articles in this Research Topic were concerned with better characterizing different aspects of autism. Li et al. used the Griffiths Mental Development Scales to characterize the cognitive, motor and social profiles of 398 autistic children (18–96 months old) in China. Findings suggested that many children showed an unbalanced profile (e.g., boys scored better than girls on eye-hand coordination, performance and practical reasoning; and differences in motor behavior became more pronounced with age). Significant aspects to take from this study were the characterization of autistic children in different regions of the world and the need to identify a child's strengths and challenges to develop personalized support.

Characterization can also be useful for predicting the future outcomes of autistic children. Forbes et al. predicted adult outcomes using an impressive dataset of participants who had been repeatedly assessed through childhood, adolescence and adulthood. Only verbal and non-verbal IQ, as well as daily living skills, could be confidently predicted from childhood data while prediction of other aspects (e.g., behavior, adult well-being, depression) was more difficult. Importantly, the authors discuss that views on what constitutes good adult outcomes for autistic children can vary. As acknowledged by the authors, this is clearly a challenging and evolving subject where stakeholder involvement is required.

## Sensory Experiences, Perception and Movement

Awareness of the significance of sensory experiences and perceptual processing on the lives of autistic individuals has increased in recent years (Torres and Donnellan, 2015; Autistica, 2016). In this Research Topic, we featured three perceptual studies that all employed rigorous, well-controlled methods to examine this topic. Mihaylova et al. used detailed psychophysical methods to progress understanding of mid-level visual processing in autistic children and adolescents. Results suggested that atypical global grouping (studied in a contour integration task), may be due to higher stimulus-dependent noise in the autistic group, leading to difficulties rejecting background noise and detecting the target.

The effect of low-mid level perceptual differences on higher level perceptual processes was elegantly shown across two studies by Lebreton et al. Here, the authors demonstrated how the commonly reported autistic preference for local compared to global detail impacted upon implicit (unconscious) and explicit (conscious) memory. This is a fascinating finding requiring replication, but has implications for understanding how perceptual style in both autistic and non-autistic individuals affects later memory recall.

Finally, Silver et al. examined whether the intense interests frequently observed in autistic individuals were related to visual processing changes for objects within that category. Contrary to expectations, there were no differences between autistic and non-autistic individuals in visual search abilities for images associated with intense interests. As such, despite enhanced time spent by autistic individuals gazing at images related to an interest, this did not seem to translate to a direct impact on visual processing ability. Linking back to Lebreton et al., we wonder whether the degree of local-global bias in the participants may mediate any relationship between visual experience and visual search ability.

In another fascinating study featured in our Research Topic, Parmar et al. conducted qualitative work with a multidisciplinary team of Optometrists, autism researchers and autistic individuals, using focus groups to provide an in-depth understanding of visual sensory issues. As well as providing a rich description of sensory experiences, the researchers highlighted how visual issues had significant negative impacts on personal well-being and daily life, but also some positive aspects (e.g., detecting details that non-autistic individuals may overlook).

Another article in our Research Topic, by Buckle et al., is the first to highlight Autistic Inertia—a debilitating difficulty of acting on intentions. The article was led by an autistic researcher (based on calls for research on this topic from autistic individuals) and the research highlighted how significant, and potentially common, Autistic Inertia is. Using qualitative methods, the study provided a detailed description of Inertia and the impact of it on autistic people's lives. Two particularly revealing findings were the benefit of other people in helping the individual to overcome being “stuck” and participants wanting to interact with others, but being unable to initiate interaction (which may be interpreted as a lack of social interest).

## Language

New approaches in the study of linguistic properties of autism were reported in this Research Topic. Marini et al. combined macrolinguistic (pragmatic, contextual processing) and microlinguistic (word and sentence processing) perspectives of language, which have traditionally been considered independently, showing that morphological and grammatical difficulties were related. Such findings suggest a relationship between difficulties in message planning and organization, which might impact children's grammatical production skills.

New avenues in language research were also highlighted by Sturrock et al. when considering potential gender differences in linguistic studies of autistic people. From a synthesis of previous literature, the authors concluded that there was a very specific profile of language and communication strengths and weaknesses for autistic females without intellectual disability, when compared to both autistic males and non-autistic females. The authors discuss how poorer recognition of autism in females might be influenced by female advantages in aspects of linguistic functioning (but see Lehnhardt et al., 2016).

In a further paper, Williams et al. demonstrated a new approach to studying communication differences between autistic and non-autistic people using relevance theory. This account posits that optimal communication is based on shared and mutually recognized relevance of utterances, which might be mismatched between autistic and non-autistic people when communicating due to differences in experiences of the world. This theoretical approach feeds into the discussions of double-empathy theory (see Theories and mechanisms).

## Support and Interventions

Leadbitter et al.'s article proposes that early intervention research could and should be aligned with principles derived from autistic self-advocacy and the neurodiversity movement. Engagement with these principles would lead to, for example, intervention research focusing on changing environments (as opposed to changing autistic people), as well as intervention researchers respecting autistic developmental trajectories and priorities for intervention targets.

In line with this approach, Di Renzo et al. examined the interactions between autistic children and their parents during play, finding that parents who were more accepting of their children's autism diagnosis and who were better able to see things from their children's perspective, were more likely to be attuned with their children during play. Such work highlights the central role of parents as partners in supporting autistic children, and the importance of shared understanding between autistic people and their non-autistic communicative partners (see section Theories and Mechanisms).

Two further studies focused on the important role of parents. Papadopoulos et al. considered support and intervention for young disabled people, 41% of whom had a primary diagnosis of autism. The authors concluded that, to ensure that organized physical activities met the needs of young disabled people, there was a need for activities to be enjoyable, for the participation of siblings and parents to be promoted, and for low-income families to be supported to participate. This work again emphasizes that autism interventions can focus on changing the structures around young people, as opposed to changing the young people themselves.

Relatedly, Devenish et al. examined the effects of lower rates of community participation by autistic young people on their caregivers. Devenish et al. found that if caregivers perceived community supportiveness to be low, this predicted caregiver feelings of isolation. Findings were interpreted within a social model of disability, highlighting how autistic people are disabled by barriers in society.

Not all intervention studies featured in this Research Topic found positive effects of interventions (moving away from the publication bias that once dominated published intervention research). Brehm et al. conducted an initial evaluation of a training programme for parents of autistic children without intellectual/language impairments. The purpose of the evaluation was to evaluate how acceptable the training was for parents, and the results were positive with hardly any parents dropping out of the training programme. Yet a variety of primary outcome measures (e.g., quality of life, social communication) did not show significant improvement. Brehm et al. note that these findings can be useful for directing future work on such interventions.

Similarly, Saul and Norbury presented an alternative to Randomized Controlled Trials for research with rare/complex populations. Drawing on a research study with minimally verbal autistic children, the authors tested the efficacy of a parent-mediated app designed to support speech production, *via* Randomization Tests and Between Case Effect Sizes. As with Brehm et al.'s study, there was no significant effect of the intervention. Yet the research still made an important contribution to the literature; notably demonstrating the importance of robust experimental design and replicable approaches, as well as showing how it is possible to conduct rigorous intervention research with rare or complex samples.

It was also encouraging to see an example of a high-quality case study featured in the article by Courchesne et al., which critically considered the role of interests and strengths in autism, particularly highlighting that these aspects do not necessarily link with academic potential. Courchesne et al. discussed an autistic teenager, C.A., who had above-average musical and calendar calculation abilities, along with pronounced difficulties in other areas (e.g., receptive and expressive language disorder). This discrepancy was found to lead to anxiety, frustration and some behavioral issues due to pressure to use his relative strengths to learn academic skills. Yet, an intervention package that focused on expectations, anxiety and emotional regulation through psychiatric intervention, parental coaching and psychotherapy, improved well-being and behavior. Courchesne et al. caution that while strengths and interests can lead to emotional well-being they should be seen as independent from adaptive outcomes such as academic achievement.

## Methods and Technologies

A key message from studies in this theme is the need to develop and validate more ecologically valid assessments of autistic characteristics. For example, Morrison et al. administered standardized measures of social cognition, social skill, and social motivation to autistic and non-autistic adults, and assessed whether these predicted “real-world” social interaction outcomes (measured using unstructured conversations with unfamiliar social partners). While autistic adults scored lower than their non-autistic peers on the three standardized social tasks and were evaluated less favorably during the unstructured social interaction, the links between performance on the standardized measures and unstructured interaction were minimal. The authors therefore question the utility of traditional measures of social performance in autistic people, calling for more ecologically valid assessments.

In line with this approach, Schaller et al. used mobile eye-tracking glasses during autism diagnostic assessments to record gaze behavior of autistic and non-autistic children and adolescents. The authors focused on the percentage of time spent looking at different areas of interest of the face and body of the interviewer and the surrounding space. Significant group differences were found, with non-autistic participants appearing to process faces and facial expressions in a holistic way focusing on the central-face region, whereas autistic participants tended to avoid this face region. The authors stress that the results are preliminary and in need of replication, but this represents an exciting avenue for further work using an ecologically valid methodology.

## Conclusions and Future Directions

Illuminating psychological science on autism from different thematic perspectives has shown several directions we can observe in the field of psychological research. For example: researchers taking a broader perspective, by incorporating previously distinct areas or methods into comprehensive studies; pairing quantitative analysis with qualitative appraisal of experience; putting forward unifying theories spanning different fields; examining an autistic person's strengths and challenges and tailoring more personalized support; developing alternative methods for evaluating interventions in more complex populations; and the implementation of a participatory approach to research. We would like to thank the contributors for their varied and stimulating contributions and hope that this Research Topic stimulates further cutting-edge psychological research that benefits the autistic community.

## Author Contributions

EG drafted a first version of the Editorial. EG, LC, and CF-W wrote sections of the manuscript. All authors contributed to manuscript revision, read, and approved the submitted version.

## Conflict of Interest

The authors declare that the research was conducted in the absence of any commercial or financial relationships that could be construed as a potential conflict of interest.

## Publisher's Note

All claims expressed in this article are solely those of the authors and do not necessarily represent those of their affiliated organizations, or those of the publisher, the editors and the reviewers. Any product that may be evaluated in this article, or claim that may be made by its manufacturer, is not guaranteed or endorsed by the publisher.
